# Liver Transplantation for HBV‐Related Disease in France: NUC Type Before LT Is Associated With Patient Survival

**DOI:** 10.1111/liv.70737

**Published:** 2026-06-11

**Authors:** Isaure Bienvenue, Olivier Roux, Audrey Coilly, Armand Abergel, Teresa Antonini, Thierry Artzner, Olivier Boillot, Faiza Chermak, Filomena Conti, Sébastien Dharancy, Christophe Duvoux, Laure Elkrief, Marwin Farrugia, Claire Francoz, Baptiste Giguet, Jean Hardwigsen, Pauline Houssel‐Debry, Marie‐Noëlle Hilleret, Nassim Kamar, Marianne Latournerie, Magdalena Meszaros, Sylvie Radenne, Bruno Roche, Ephrem Salamé, Delphine Verhoeven‐Weil, Didier Samuel, Fabien Zoulim, Jérôme Dumortier, François Villeret

**Affiliations:** ^1^ Service d'Hépatologie et de Transplantation Hépatique Hospices Civils de Lyon, Université Claude Bernard Lyon 1, INSERM Unit 1350 PathLiv, Lyon Hepatology Institute, IHU EVEREST Lyon France; ^2^ Service d'Hépatologie et Transplantation Hépatique Hôpital Beaujon, APHP Clichy France; ^3^ Centre Hépato‐Biliaire, Hopital Paul Brousse, Assistance Publique Hôpitaux de Paris Unité Inserm 1193, Université Paris‐Saclay Villejuif France; ^4^ Département de Médecine Digestive CHU Estaing Clermont‐Ferrand France; ^5^ Service de Chirurgie Hépato‐Bilio‐Pancréatique et Transplantation Hépatique CHRU Hautepierre Strasbourg France; ^6^ Fédération des Spécialités Digestives Hospices Civils de Lyon, Hôpital Edouard Herriot, IHU EVEREST Lyon France; ^7^ Service de Chirurgie Hépatobiliaire et de Transplantation Hépatique CHU Haut Lévêque Bordeaux France; ^8^ Service de Chirurgie Digestive, Hépato‐Biliaire et de Transplantation Hépatique Hôpital Pitié Salpêtrière, AP‐HP Paris France; ^9^ Service d'Hépatologie Hôpital Claude Huriez, CHRU Lille Lille France; ^10^ Service d'Hépatologie Hôpital Henri Mondor, APHP Créteil France; ^11^ Service de Chirurgie Digestive, Oncologique et Transplantation Hépatique Hôpital Trousseau, CHU Tours Tours France; ^12^ Service de Chirurgie Digestive et de Transplantation Hépatique CHU Archet II Nice France; ^13^ Service d'Hépatologie et Transplantation Hépatique Hôpital Universitaire de Pontchaillou Rennes France; ^14^ Service Chirurgie Générale et Transplantation Hépatique Hôpital La Timone, APHM Marseille France; ^15^ Service d'Hépato‐Gastro‐Entérologie CHU Michallon Grenoble France; ^16^ Département de Néphrologie et Transplantation d'Organes CHU Rangueil Toulouse France; ^17^ Service d'Hépatologie et de Gastro‐Entérologie CHU Dijon‐Bourgogne Dijon France; ^18^ Département d'Hépatologie et Transplantation Hépatique CHU Saint Eloi Montpellier France; ^19^ Service d'Hépatologie et Soins Intensifs Digestifs Hôpital Jean Minjoz Besançon France

**Keywords:** HBIG, HBV, HCC, liver transplantation, NUC

## Abstract

**Background & Aims:**

Liver transplantation (LT) is indicated for liver complications related to hepatitis B virus (HBV) infection: acute liver failure (ALF), decompensated cirrhosis or hepatocellular carcinoma (HCC). The present study aimed to describe and evaluate patient survival after LT for HBV‐related disease in France and identify the factors influencing survival.

**Methods:**

The present retrospective cohort study based on medical record information included all adult patients transplanted with positive HBsAg (+/− coinfection with Hepatitis D virus (HDV)) between January 1, 2005 and December 31, 2023 in all French LT centres.

**Results:**

The study population consisted of 1083 patients, the majority of whom were men (81.5%) with a median [IQR] age at LT listing of 52.8 [42.5–59.8] years. Indications for LT were HBV‐related HCC (47.2%), HBV‐related cirrhosis (28.5%), HDV‐related cirrhosis (11.2%), HBV‐related ALF (10.5%), HDV‐related HCC (1.4%) and other (0.7%). Median [IQR] post‐LT follow‐up was 6.0 [2.2–11.1] years. Patient survival at 1, 5, 10 and 15 years after LT was 91.6%, 80.1%, 71.8% and 63.6% respectively. Multivariate analysis showed that independent significant prognostic factors were age at LT (HR: 1.030; 95CI: 1.019–1.042; *p* < 0.0001) and the pre‐LT nucleos(t)ides analogue (NUC) regimen: in comparison to tenofovir, the use of entecavir alone (HR: 1.735; 95CI: 1.312–2.295; *p* < 0.0001) or another NUC or combination therapy (HR: 1.471; 95CI: 1.059–2.043; *p* = 0.021) were associated with decreased survival.

**Conclusions:**

Survival after LT for HBV‐related liver disease is good. NUC type prior to LT seems to be associated with patient survival.

## Abstract (highlights)

**Key Points:**

- HCC is the leading indication for LT in France.
- Patient survival is estimated at 71.8% at 10 years.
- The NUC type used prior to LT is associated with post‐transplant survival.

**Lay Summary:**

- Main indication for LT in France in the context of HBV infection is HCC.
- Overall patient survival after LT is good, estimated at 80.1%, 71.8% and 63.6% at 5, 10 and 15 years, respectively.
- Patients treated with tenofovir before LT have better survival rates than those treated with entecavir or other NUCs.

**Abbreviations**
ACLF: acute on chronic liver failure
AFP: alpha foeto‐protein
ALF: acute liver failure
AZA: azathioprine
BMI: body mass index
cccDNA: covalently closed circular DNA
CHB: chronic hepatitis B
CNI: calcineurin‐inhibitor
CYA: cyclosporine
DNA: deoxyribonucleic acid
ELTR: European Liver Transplant Registry
HBIG: hepatitis B immunoglobulins
HBV: hepatitis B Virus
HCC: hepatocarcinoma carcinoma
HCV: hepatitis C Virus
HIV: human immunodeficiency virus
INR: international normalized ratio
LLoD: lower limit of detection
LT: liver transplantation
mm: millimetresmillimeters
MMF: mycophenolate mofetil
mTor‐i: mTor inhibitor
NUC: Nucleos(t)ide analogues
SMD: standardized mean differences
TAC: tacrolimus

## 1 Background and Aims

Chronic hepatitis B (CHB) affects 254 million people worldwide and represents a major cause of morbidity and mortality due to severe complications such as liver cirrhosis and hepatocellular carcinoma (HCC) [1]. Hepatitis B Virus (HBV) is responsible for chronic inflammation, resulting in the long‐term development of chronic hepatitis, cirrhosis and HCC (with or without underlying cirrhosis). HBV can also be responsible for acute hepatitis in cases of primary infection or reactivation (notably during immunosuppressive treatment without prior appropriate prophylactic antiviral therapy). In addition, HBV infection can be associated with hepatitis D virus (HDV) co‐infection. This co‐infection usually results in more severe and rapidly progressing chronic liver disease [2].

HBV is a minor cause of chronic liver disease in France, compared with Asian countries where the prevalence of HBV is much higher in the general population [3]. Current CHB treatments, based on nucleos(t)ide analogues (NUC), are effective in decreasing serum HBV DNA, but are curative in < 5% of cases due to the persistence of intrahepatic covalently closed circular DNA (cccDNA) and HBV genomic integration [4, 5, 6]. Despite therapeutic advances and the recent use of bulevirtide, the natural history of HDV‐HBV co‐infection remains severe, with a high incidence of HCC and cirrhosis [2]. Liver transplantation (LT) represents the definitive treatment for decompensated HBV+/−HDV cirrhosis, HCC and acute liver failure (ALF). After LT, a combined prophylactic regimen based on NUC and Hepatitis B immunoglobulins (HBIG) is highly efficient and recommended by most international liver societies [7, 8, 9].

A large 2013 European study reported that the survival rate of patients who have undergone LT for HBV+/−HDV infection was similar to that for other LT indications: overall survival was estimated at 68% at 10 years [6]. Nevertheless, few studies have analysed the risk factors for mortality, with the majority focusing on the risk factors for HBV recurrence after LT [10, 11].

The present nationwide retrospective study aimed to evaluate from a recent and large cohort of patients the overall survival after LT for HBV‐related disease and identify factors influencing survival.

## 2 Patients and Methods

### 2.1 Study Population

We included all adult patients transplanted in all French LT centres, based on the national database of the French Agence de la Biomédecine (ABM) and local databases. We selected all patients transplanted between January 1st 2005 and December 31st 2023 for “HBV cirrhosis”, “HDV cirrhosis”, “HCC” and “Acute liver failure” in participating French centres. We reviewed all medical records and selected only patients with a positive HBsAg at the time of LT. Exclusion criteria were HCV co‐infected patients with a positive viral load at the time of LT. Patients with Human Immunodeficiency Virus (HIV) coinfection were included regardless of the serum HIV viral load. Patients with chronic liver comorbidities (history of alcohol consumption, metabolic syndrome, etc.) were included in the study if they were HBsAg positive at LT time. This study was conducted in accordance with the Declaration of Helsinki. According to French law (*Loi Jardé*), retrospective studies do not require Institutional Review Board approval.

### 2.2 Clinical and Biological Characteristics at LT Listing and at LT

To retrieve the data, we asked each centre to extract data from the French national transplantation database Cristal. Information not available in the Cristal database was retrieved from computerized or paper patient records.

Reasons for registration on the LT list were collected: cirrhosis (Acute on Chronic Liver Failure (ACLF) or not), HCC or ALF. Virological parameters at the time of LT listing were collected: serum HBV DNA, serum HDV RNA, quantitative HBsAg, HBe status, type of NUC, treatment of HDV infection, co‐infection with HIV and/or HCV. The serum HBV DNA quantification thresholds during the study period ranged from 10 IU/mL to 2000 copies/mL; all results were converted to IU/mL. Cirrhosis characteristics at LT listing and at LT time were specified with the MELD score and the CHILD‐PUGH score. The presence of HCC at enrolment and its characteristics (number of nodules, size of the largest nodule and AFP rate) were retrieved and taken into account when calculating the AFP score.

### 2.3 Follow‐Up After LT

Patients received grafts from cadaveric or living donors. Initial immunosuppressive regimen was based on a calcineurin‐inhibitor (CNI): cyclosporine (CYA) or tacrolimus (TAC). Induction therapy by polyclonal antibodies or anti‐interleukin‐2 receptor antibodies was mainly administered in case of acute kidney injury. Starting on postoperative day 1, methylprednisolone was tapered to reach a maintenance dose of 0 to 5 mg/day at 6 months post‐transplantation. Azathioprine (AZA) or mycophenolate mofetil (MMF) or sirolimus/everolimus (mTor inhibitor (mTor‐i)) were either administered as part of an initial triple immunosuppressive regimen or introduced during follow‐up as maintenance immunosuppressive agents. Outpatient follow‐up visits were usually conducted once a week during the first month after discharge from the hospital, twice a month during the second and third months, monthly for the rest of the first year, and every 3 or 4 months thereafter, regardless of post‐LT observation period length. Additional visits were made when necessary. A complete laboratory panel, including haematology, liver parameters, coagulation, electrolytes, total protein, renal parameters and blood CNI trough levels or mTor‐i levels, was conducted at each visit. The end of follow‐up corresponded to death, the last medical examination or date of loss of follow‐up. All data were retrospectively collected until December 31st 2024.

### 2.4 HBV Prophylaxis and HBV Recurrence After LT

All patients received HBIG prophylaxis during LT and for at least 5 days thereafter. Prophylaxis of post‐LT HBV recurrence was collected: NUC regimen, HBIG perfusion (route of administration, intravenous or subcutaneous and duration). Post‐LT HBV recurrence was defined by a positive serum HBV DNA. During the study period, the lower limit of detection (LLoD) for serum HBV DNA ranged from 2 to 2000 IU/mL, depending on the year and centre.

Concerning NUC treatment, the “tenofovir” category includes all patients receiving treatment with tenofovir disoproxil or tenofovir alafenamide (as monotherapy or in combination for HIV infection). The “other” category includes NUCs other than tenofovir and entecavir. Patients treated with tenofovir/entecavir combination before LT were excluded from statistical analyses.

### 2.5 Statistical Analysis

All data were analysed using SPSS software, version 23.0 (IBM, Armonk, NY, US). Data were described in their totality using median with interquartile range [IQR] or mean with standard deviation (SD) for continuous variables and number (percentage) for categorical variables. Categorical variables were compared with the Chi‐square or Fischer's exact tests, and quantitative variables were compared using the Student *t*‐test or non‐parametric tests (Mann–Whitney or Kruskall–Wallis tests) when appropriate. Patient survival was calculated from the date of LT to date of death or the last clinical visit. Graft survival was calculated from the date of LT to date of liver re‐transplantation (re‐LT), death or last visit if no re‐LT. Survival curves were constructed with the Kaplan–Meier method and compared with the log‐rank test in univariate analysis. The Cox proportional hazards regression model was used in multivariate models. All significant variables in the univariate analysis with a level set at *p* < 0.1 were incorporated into multivariate models (by excluding collinear variables (defined according to Pearson correlations)). To account for the effect of the LT period and investigate any potential impact of the LT period, we divided the study period into four distinct periods: 2005–2009; 2010–2014; 2015–2019; and 2020–2023. To investigate a potential centre effect in this multicentre study, we divided the LT centres into 4 categories based on the number of LTs performed during the study period (divided according to the median and quartiles of LTs number over the study period: < 30; 30–38; 38–66; ≥ 66).

We conducted a stratified analysis using propensity score quintiles to account for potential confounding in the comparison between patients treated with tenofovir or entecavir at LT time. The propensity score was estimated via logistic regression, incorporating the following baseline covariates: LT period (divided into 4 categories), LT centres (divided into 4 categories based on the number of LTs performed during the period), age at LT, gender, LT indication (cirrhosis, hepatocellular carcinoma [HCC] or acute liver failure [ALF]), MELD score at LT time, CHILD‐Pugh score at LT time, HDV‐coinfection and body mass index (BMI) at LT. Patients were then divided into seven equally sized strata based on their propensity scores.

Standardized mean differences (SMD) were calculated for all covariates before and after propensity score stratification. Covariate balance was assessed using SMD, with values < 0.1 considered indicative of good balance. This propensity score stratification approach ensured comparability between patients treated with tenofovir and entecavir at LT time within each propensity score stratum, thereby reducing the risk of confounding bias in the analysis of treatment outcomes [12]. A *p* value less than 0.05 was considered statistically significant. We analysed the “cirrhosis” and “HCC” groups separately, while also calculating a propensity score, using the same methodology and quality criteria. However, when calculating the propensity score, we excluded the aetiology of LT.

## 3 Results

### 3.1 Population Characteristics (Table 1)

**TABLE 1:** Patients characteristics at LT listing.

General characteristics at LT listing	
Age (years; IQR)	52.8 [42.5; 59.8]
Recipient gender M/F (patients, %)	883 (81.5%)/200 (18.5)
BMI (kg/m^2^) (median; IQR)	24.9 [22.5–27.8]
Overweight (patients, %)	522 (48.2)
Obesity (all grades) (patients, %)	141 (13.0)
Consumption of alcoholic beverages (patients, %)	107 (9.9)
CHILD‐PUGH score (median; IQR) (total of patients)	B8 [A5; C11]
CHILD‐PUGH A (patients, %)	396 (36.6)
CHILD‐PUGH B (patients, %)	252 (23.3)
CHILD‐PUGH A (patients, %)	435 (40.2)
MELD score (median; IQR) (total of patients)	13.3 [7.62; 24.4]
Renal dialysis (patients, %)	33 (3.1)
HCC (yes/no) (patients, %)	483 (45.9)/556 (54.1)
Median serum AFP level (IQR) (μg/L)	6 [3–17]
Median number of HCC lesions (IQR)	1 [1–2]
Size of the largest HCC lesion (IQR) (mm)	42 [36–50]
Virological characteristics at LT listing	
HBV DNA—not detectable (patients, %)	630 (63.4)
HBV DNA—Detectable non‐quantifiable (patients, %)	70 (7.0)
HBV DNA—Quantifiable (patients, %)	294 (29.6)
If HBV DNA quantifiable, median level (LogIU/mL; IQR)	3.9 [2.11; 6.29]
HBeAg (positive/negative) (patients, %)	117 (18.2)/525 (81.8)
HDV positive serology (yes/no) (patients, %)	273 (26.3)/767 (73.7)
HDV RNA detectable (patients, %)	103 (9.5)
HDV RNA median level (LogU/mL; IQR)	5.3 [4–6.9]
HIV co‐infection (yes/no) (patients, %)	64 (5.9)

Abbreviations: AFP, alpha‐fetoprotein; BMI, body‐mass index; DNA, deoxyribonucleic acid; HBV, hepatitis B; HCC, hepatocellular carcinoma; HDV, hepatitis D; HIV, human immunodeficiency virus; IQR, interquartile range; LT, liver transplantation; MELD, model for end‐stage liver disease; RNA, ribonucleic acid.

One thousand eighty‐three patients transplanted between January 1, 2005 and December 31, 2023 were included. The median [IQR] number (57 [50–62]) of LTs remained stable during this period. The population was majority male (81.5%, 833/1083) with a median age at LT of 52.8 years [42.5–59.8]. In descending order, LT indications were: HBV‐related HCC (47.2%; 511/1083), HBV‐related cirrhosis (28.5%; 309/1083), HDV‐related cirrhosis (11.6%; 126/1083), HBV‐related ALF (10.5%; 114/1083), HDV‐related HCC (1.4%; 15/1083) and other (0.7%; 7/1083). Four hundred nighty‐eight (45.9%; 498/1083) patients had HCC at LT listing. At LT listing, the majority of patients (79.9%; 398/498) had an AFP score inferior or equal to 2. Twenty‐six patients (2,4%; 26/1083) received a combined transplant: 21 liver‐kidney transplants and 5 liver‐heart transplants. The characteristics of patients who underwent LT for cirrhosis and HCC are presented in Table S1.

At the time of LT, 411 patients were treated by tenofovir (40.9%, 411/1004 patients with data available), 347 by entecavir (34.6%; 347/1004), 143 were receiving another NUC treatment (14.2%; 143/1004), 76 patients were not receiving antiviral treatment (7.6%; 76/1004), and 27 patients were receiving both tenofovir and entecavir (2.7%; 27/1004). NUC therapy prior to LT was not available for 79 patients (7.3%; 79/1083). Among patients receiving another type of NUC, 64 were on lamivudine (44.8%; 64/143), 54 on dual therapy with adefovir and lamivudine (37.8%; 54/143), 21 on adefovir alone (14.7%; 21/143) and 4 on another treatment (2.8%; 4/143). At the time of LT, there was a difference in age (*p* = 0.001) between patients receiving tenofovir and those receiving entecavir (Table 2). At LT, the majority of patients had an undetectable serum HBV DNA (75.9%; 735/969 patients with HBV DNA serum available), 43 patients had a detectable but not quantifiable (DNQ) serum HBV DNA (4.4%; 43/969) and 191 patients, mostly with ALF, had quantifiable serum HBV DNA (19.7%; 191/969). Among patients with quantifiable serum viral load at LT, median serum HBV DNA was 3.48 [2.1–6.3] LogIU/mL. One hundred fourteen patients did not have HBV DNA results available before LT (10.5%; 114/1083).

**TABLE 2:** Comparison of populations between the two main types of NUCs at LT time.

	NUC type at LT time		Univariate analysis (*p*)
	Tenofovir (*n* = 411)	Entecavir (*n* = 347)	
Treatment duration before LT (median, IQR)	2.85 [0.86–6.50]	2.90 [0.94–5.99]	0.654
LT period (4 categories) (patients, %)	34 (8.3)/96 (23.4)/154 (37.5)/127 (30.9)	30 (8.6)/108 (31.1)/116 (33.4)/93 (26.8)	0.102
Gender (Male/female) (patients, %)	327 (79.4)/84 (20.4)	281 (80.7)/66 (19.0)	0.346
Etiologies (Cirrhosis, HCC, ALF) (patients, %)	179 (43.4)/198 (48.1)/34 (8.3)	138 (38.6)/181 (52.2)/32 (9.2)	0.199
Age (median, IQR)	52 (42–59)	55 (45–63)	**0.001**
MELD score (median, IQR)	15 (8–25)	15 (7–24)	0.720
CHILD score (median, IQR)	B8 (A5‐C11)	B8 (A5‐C11)	0.353
Serum creatinine (median, IQR)	78 (64–97)	77 (62–98)	0.921
HCC at LT listing (yes/no) (patients, %)	218 (48.8)/193 (48.8)	175 (50.4)/172 (49.6)	0.099
BMI at LT listing (median, IQR)	25 (23–28)	25 (22–28)	0.523
HBV DNA at LT listing (undetectable/detectable) (patients, %)	342 (83.0)/69 (16.7)	290 (83.6)/57 (16.4)	0.475
HDV coinfection (Yes/No) (patients, %)	62 (15.0)/349 (84.7)	37 (10.7)/310 (89.3)	0.492

Abbreviations: ALF, acute liver failure; BMI, body‐mass index; DNA, deoxyribonucleic acid; HBV, hepatitis B; HCC, hepatocellular carcinoma; HDV, hepatitis D; LT, liver transplantation; MELD, model for end‐stage liver disease; NUC, nucleos(t)ide analogues.

### 3.2 Recurrence of HBV Infection

In the early post‐LT phase, all patients received HBIG prophylaxis for at least 5 days. Most patients remained on the same NUC therapy before and immediately after LT: among patients treated with tenofovir before LT, 3 switched to entecavir after LT; among patients on entecavir before LT, 21 switched to tenofovir after LT. Among patients without NUC treatment before LT, 61/76 (80.3%; 61/76) started NUC therapy post‐LT (29 entecavir, 25 tenofovir and 7 lamivudine), 6/76 (7.9%; 6/79) received no NUC treatment and no information was available for 9/76 (11.8%; 9/76) patients. Upon discharge after LT, 478 patients (44.1%; 478/1083) were treated with HBIG+NUC combination therapy, 449 patients (41.5%; 449/1083) with NUC alone, 18 patients (1.7%;18/1083) were treated with HBIG alone, 77 patients (7.1%;77/1083) patients died before discharge and data were unavailable for 61 patients (5.6%; 61/1083). Twenty‐two patients developed HBV reactivation during follow‐up, with a median delay of 2.6 [0.7–3.2] years. The cumulative incidence of HBV reactivation after LT was estimated at 0.5%, 1.9%, 3.6% and 4.1% at 1, 5, 10 and 15 years, respectively.

### 3.3 Patient and Graft Survival (Figure 1)

**FIGURE 1:**
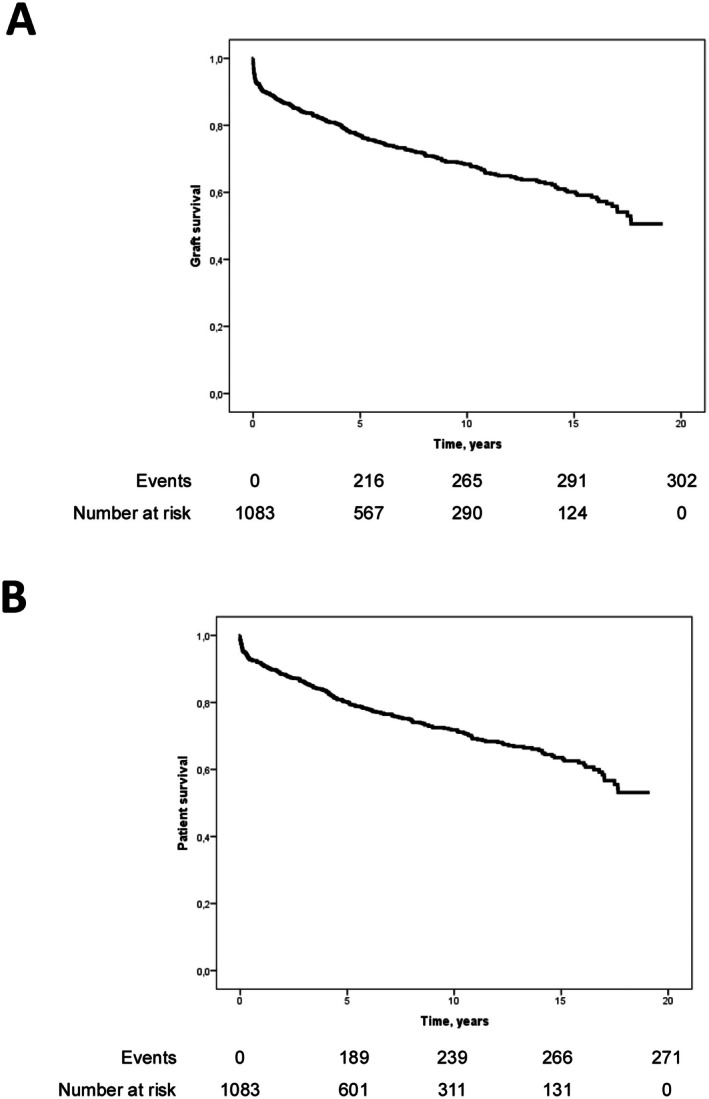
Graft survival (A) and overall patient survival (B) (according to Kaplan–Meier estimates). (A) Graft survival at 1, 5, 10 and 15 years after LT was 88.4%, 77.4%, 69.0% and 60.8%, respectively. (B) Patients' survival at 1, 5, 10 and 15 years after LT was 91.6%, 80.1%, 71.8% and 63.6%, respectively.

Median post‐LT follow‐up was 6.0 [2.2–11.1] years. Graft survival at 1, 5, 10 and 15 years after LT was 88.4%, 77.4%, 69.0% and 60.8%, respectively (Figure 1A). Sixty‐eight re‐LT occurred during follow‐up, with a median delay of 21 days [3–325]. The causes of re‐transplantation were: artery thrombosis (47.1%; 32/68), primary non‐function (33.8%; 23/68), chronic rejection (4.4%; 3/68), biliary complications (4.4%; 3/68) and other (10.3%; 7/68). No re‐LT was associated with HBV reinfection.

**FIGURE 2:**
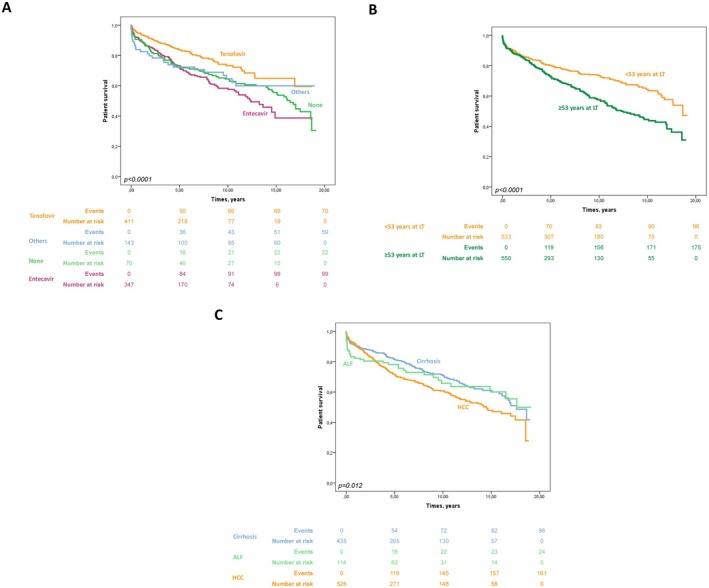
Factors associated with overall patient mortality in univariate analysis (according to Kaplan–Meier estimates). (A) Overall patient survival according to pre‐LT NUC type (*p* < 0.0001): *Tenofovir*: Patient survival at 1, 5, 10 and 15 years after LT was 94.3%, 83.9%, 73.3% and 64.9%, respectively. *Entecavir*: Patient survival at 1, 5, 10 and 15 years after LT was 89.4%, 71.6%, 58.4% and 38.7%, respectively. *Other NUC*: Patient survival at 1, 5, 10 and 15 years after LT was 89.9%, 72.5%, 64.4% and 54.6%, respectively. *None*: Patient survival at 1, 5, 10 and 15 years after LT was 82.5%, 72.3%, 62.3% and 59.9%, respectively. (B) Overall patient survival according to patient age at LT (53 years, median age of transplant patient) (*p* < 0.0001). *Patients with age ≤ 53 years at LT time*: Patient survival at 1, 5, 10 and 15 years after LT was 93.0%, 85.0%, 80.8% and 74.4%, respectively. *Patients with ≥ 53 years at LT time*: Patient survival at 1, 5, 10 and 15 years after LT was 90.0%, 75.4%, 63.2% and 51.9%, respectively. (C) Overall patient survival according to aetiology of LT (cirrhosis, HCC, ALF) (*p* = 0.012). *Patients transplanted for decompensated cirrhosis*: Patient survival at 1, 5, 10 and 15 years after LT was 95.3%, 85.6%, 77.1% and 72.7%, respectively. *Patients transplanted for HCC*: Patient survival at 1, 5, 10 and 15 years after LT was 92.2%, 73.7%, 65.1% and 55.6%, respectively. *Patients transplanted for ALF*: Patient survival at 1, 5, 10 and 15 years after LT was 87.4%, 84.7%, 75.6% and 73.0%, respectively.

Overall patient survival at 1, 5, 10 and 15 years after LT was 91.6%, 80.1%, 71.8% and 63.6%, respectively (Figure 1B). Sixty‐one (5.6%; 61/1083) patients were lost to follow‐up during the study. In patients transplanted for HCC, overall survival at 1, 5, 10 and 15 years was 92.2%, 73.7%, 65.1% and 55.6%, respectively (Figure 2C). In patients transplanted for ALF, survival at 1, 5, 10 and 15 years was 87.4%, 84.7%, 75.6% and 73.0%, respectively. In transplant patients with decompensated cirrhosis, survival at 1, 5, 10 and 15 years was 95.3%, 85.6%, 77.1% and 72.7%, respectively (Figure 2C).

Two hundred seventy‐one patients died during follow‐up with a median time from LT of 3.1 [0.5–7.2] years. The main causes of death after LT were HCC recurrence (28.4%; 77/271), infection (24.0%; 65/271), oncological causes excluding HCC recurrence (10.3%; 28/271); 28 patients (10.3%; 28/271) died immediately after LT, 16 (5.9%; 16/271) died secondary to cardiovascular disease, 11 (4.1%; 11/271) related to general medical causes, 7 (4.1%; 7/271) related to graft rejection, 5 (1.8%; 5/271) secondary to an accident, 10 (3.7%; 10/271) for other reasons and 24 (8.9%; 24/271) from unknown causes. No deaths were associated with HBV reactivation.

### 3.4 Risk Factors Associated With Patient Death (Table 3; Figure 2)

**TABLE 3:** Pre‐LT risk associated to overall death after LT.

	Univariate analysis (*p*)	Multivariate analysis	
		HR (95% CI)	*p*
LT period (4 period)	0.639		
Liver Transplant Center (4 categories)	0.863		
Gender	0.208		
Alcohol consumption before LT	0.172		
HCV positive serology	0.284		
LT indication (cirrhosis, ALF, HCC)	**0.012**	1.040 (0.869–1.246)	0.667
Cirrhosis at LT time (yes/no)^a^	**0.006**		
ALF at LT time (yes/no)^a^	**0.006**		
HCC at LT time^a^	**0.001**		
Age at LT time	**0.0001**	**1.030 (1.019–1.042)**	**< 0.0001**
Age ≥ 53 years (yes/no)	**0.0001**		
MELD score at LT time^a^	**0.0011**		
CHILD‐PUGH score at LT time^a^	**0.0001**		
Serum creatinine at LT time^a^	**0.0001**		
Albumin at LT time^a^	**0.029**		
Total bilirubin at LT time^a^	**0.025**		
INR at LT time^a^	**0.002**		
Natremia at LT time^a^	0.144		
AFP score (< 2 vs. ≥ 2)	0.494		
Overweight	0.144		
Obesity (all grades)	0.313		
HBV DNA at LT time (undetectable/detectable)	0.490		
HBV DNA at LT time (quantifiable versus non‐quantifiable)	0.298		
HBeAg at LT time (pos/neg)	0.724		
HDV coinfection (Yes/No)	0.244		
Pre‐LT NUC type	**0.0001**		
Tenofovir		**1**	
Entecavir		**1.735 (1.312–2.295)**	**< 0.0001**
Others NUCs		**1.471 (1.059–2.043)**	**0.021**
None		1.521 (0.967–2.394)	0.070

*Note:* The two values shown in the third column are the 95% confidence interval (95% CI).

Abbreviations: ALF, acute liver failure; BMI, body‐mass index; DNA, deoxyribonucleic acid; HBV, hepatitis B; HCC, hepatocellular carcinoma; HDV, hepatitis D; INR, international normalized ratio; LT, liver transplantation; MELD, model for end‐stage liver disease; NUC, nucleos(t)ide analogues.

^a^ Due to multicollinearity, the multivariate analysis model only took into account uncorrelated variables: Several variables were collinear with the LT indication: MELD score, CHILD score, biological parameters taken into account by these scores. We chose the LT indication for the multivariate model.

Among the parameters available prior to LT, the following were significantly associated with patient survival in univariate analysis: LT indication (cirrhosis, ALF or HCC), age at listing, MELD score at time of LT, CHILD score at time of LT, biological parameters included in MELD/CHILD scores (creatinine, albumin, total bilirubin levels and International Normalized Ratio (INR)) and pre‐LT NUC regimen. Serum HBV DNA (undetectable/detectable) at LT listing or HDV coinfection did not affect patient survival in univariate analysis. In multivariate analysis, age at LT (HR: 1.030; 95CI: 1.019–1.042; *p* < 0.0001) and pre‐LT NUC regimen, entecavir (HR: 1.735; 95CI: 1.312–2.295; *p* < 0.0001) or another NUC (HR: 1.471; 95CI: 1.059–2.043; *p* = 0.021) compared to tenofovir, were independently and significantly associated with overall mortality. When considering only patients who underwent LT for cirrhosis (Table S2) or for HCC (Table S3), multivariate analysis revealed an association between the use of tenofovir prior to transplantation and overall survival after LT.

After propensity score stratification, tenofovir compared to entecavir is associated with increased overall survival (OR: 1.5691; 95CI: 1.113–2.213; *p* = 0.010) in all patients. We also performed a propensity score stratification analysis separately for patients who underwent LT for primary indications of “cirrhosis” or “HCC”. After stratification by propensity score (using the same quality data as in the overall analysis, divided into 5 quintiles), we confirmed an association between tenofovir prior to LT and patient survival only in the “HCC” group (OR: 1.767; 95% CI: 1.153–2.710; *p* = 0.009). In the “cirrhosis” group, this association was not confirmed by the propensity score (OR: 1.480; 95% CI: 0.763–2.977; *p* = 0.271).

## 4 Discussion

Our large retrospective French study, with long follow‐up, confirms good patient and graft survival after LT for HBV‐related disease including ALF, cirrhosis and HCC. No deaths or re‐LT were linked to HBV reactivation. Age at LT and pre‐LT NUC regimen were associated with an impact on overall patient survival (with a trend towards reduced survival in patients not treated with tenofovir prior to LT).

In our study, patient survival at 5, 10 and 15 years after LT was estimated at 80.1%, 71.8% and 63.6%, respectively. These data are similar to data reported in the literature. In the European Liver Transplant Registry (ELTR) data published in 2013, overall patient survival was 74% and 67% at 5 and 10 years [6]. This study included patients transplanted between 1988 and 2010 and did not include ALF, which may explain the slightly lower survival rates compared to our study. In addition, among the 5912 patients who underwent LT for HBV‐related liver disease, the proportion of patients with HCC in the ELTR data was low (22% compared to 47% in our study), indicating that the indications for LT in HBV‐related liver disease have changed between this older cohort (1988–2010) and ours (2005–2023). The decrease in the proportion of patients undergoing LT for HBV‐related decompensated cirrhosis in our study compared to the ELTR data (78% in ELTR study versus 40.1% in our study) was probably linked to the advent of the latest generation of NUCs which achieve virological suppression, reduce the risk of progression towards fibrosis and cirrhosis and reduce the risk of viral reactivation after LT. The change in LT indications may also explain the variations in survival among these patients. Furthermore, as our study is more recent, improved post‐LT resuscitation conditions and immunosuppressive treatments have also led to improved survival. In a recent Italian study (including 1205 patients transplanted between 2010 and 2021, of whom 60.8% were transplanted for HCC), overall survival was slightly better than in our study (83.5% at 5 years); this better survival rate could be explained by the fact that the study began in 2010 (compared to 2005 in our case), with improved patient access to the latest generation of NUCs [13]. Furthermore, the proportion of patients transplanted for ALF was lower in the Italian study (5.0% compared to 10.5% in our study), which may explain a better early survival at 1 and 5 years in the Italian study. In a meta‐analysis including 7897 patients (mainly Asian), overall patient survival was 95.6%, 86.4% and 86.4% at 1, 5 and 10 years [14], however, this study did not include patients who had also undergone LT for ALF, which may explain the high early survival rates.

Considering risk factors available at LT, only age at LT and pre‐LT NUC regimen (with a tendency for entecavir or other NUCs compared to tenofovir to have a negative impact) were associated with an improved overall patient survival in our study. The age of the recipient has been identified as a risk factor for mortality in several LT studies regardless of the initial liver disease leading to LT. Age at LT impacts long‐term survival in these patients, particularly for those transplanted after age 60 [15]: Survival in patients transplanted after age 60 is 10 to 20% lower than in younger patients [16]. HBV DNA at LT was not considered a risk factor for overall mortality in our study. Conversely, in the ELTR study, though 71% of patients transplanted for HBV‐related liver disease had undetectable serum HBV DNA, detected HBV DNA at the time of LT was considered a risk factor for mortality or graft loss [6]. However, HBV DNA quantification standards were undetermined in the ELTR cohort and quantifiable thresholds were likely much higher than those currently available. In our study, the availability of NUCs enabling significant virosuppression (HBV DNA detection threshold between 10 and 20 IU/mL) explains the absence of impact of HBV DNA at LT on overall survival.

The second factor influencing patient survival was the type of NUC used before LT (and thereafter in the vast majority of patients). Survival of patients treated with tenofovir seemed to be better in comparison to entecavir or other NUCs across the entire cohort. This effect is also observed in the multivariate analysis of the “HCC” and “cirrhosis” subgroups. This potential benefit of tenofovir persists even after propensity score stratification in the entire cohort, when comparing patients treated with tenofovir or entecavir. Propensity score stratification helps reduce the risk of confounding factors associated with this historical cohort. This benefit of tenofovir over other NUCs was independent of HBV DNA at LT, duration of NUC treatment before LT, presence or not of HCC, MELD/CHILD score at LT and the patient's age at LT. Stratification analysis by propensity score for the “HCC” and “cirrhosis” subgroups confirms the potential benefit of tenofovir only in the “HCC” group, likely due to a lack of statistical power in the “cirrhosis” group and a lower number of deaths in the cirrhosis group. Several mechanisms of action have been found to explain this trend towards improved survival in patients treated with tenofovir for HBV‐related HCC: TDF‐treated patients exhibit elevated levels of serum interferon (IFN)‐λ3 (a factor known to have the potential to directly or indirectly inhibit tumour growth) [17], TDF restores the function of T cells and natural killer cells by downregulating interleukin (IL)‐10 and upregulating IL‐12 [18] and inhibiting the translocation of Akt to the plasma membrane [19]. Due to the retrospective nature of this study, we do not have data on possible viral resistance prior to LT; however, there is no significant difference between the two groups in terms of serum HBV DNA at LT. The majority of patients received the same NUC before and after LT, which explains this long‐term effect: 3 patients who were treated with tenofovir prior to LT switched to entecavir after LT, and 21 patients on entecavir switched to tenofovir after LT. To our knowledge, no previous study has evaluated the impact of pre‐LT NUC regimen on survival in patients who have undergone LT for HBV‐related disease. In non‐transplant patients, several cohort studies have found that tenofovir offers an advantage over entecavir in terms of overall survival [20, 21, 22, 23, 24]. Furthermore, LT patients treated with entecavir have a lower survival rate than patients treated with tenofovir, but also than patients treated with another type of NUC (such as lamivudine). Despite various analyses and the consideration of potential confounding factors, no explanation has been found for this survival advantage of tenofovir in transplant patients.

Our study is retrospective and therefore exposed to numerous biases. All files were reviewed at each participating site, thereby minimizing the lack of information. This retrospective cohort study was unable to analyse the intrahepatic virological compartment and determine intrahepatic (and subclinical) HBV recurrence [25, 26]. Due to the retrospective nature of our work, certain parameters such as treatment adherence, potential HBV mutations conferring resistance to entecavir or the ethnicity of patients were not available. This retrospective study does not allow us to conclude that there is a causal relationship between pre‐LT NUC regimen and post‐LT survival; however, it does find a statistically significant association. This association must be confirmed by prospective, randomized studies with long‐term follow‐up.

In conclusion, our large retrospective French study analysing patient survival confirms excellent survival after LT for HBV‐related disease. The potential association between pre‐LT NUC regimen and patient survival should be investigated in prospective cohorts.

## Author Contributions

Jérôme Dumortier and François Villeret conceived the project and participated in the analysis, interpretation of data and are guarantors of the article. Isaure Bienvenue collected the data. François Villeret, Isaure Bienvenue and Jérôme Dumortier performed statistical analysis. Isaure Bienvenue, François Villeret and Jérôme Dumortier participated in writing of the manuscript. All authors were involved in medical care of the patients, reviewed and approved the final version of the manuscript.

## Funding

Grant from Grifols France (for travel and accommodation expenses incurred for data collection in the participating centres).

## Conflicts of Interest

O.R.: Gilead (consultant/travel fees)/Chiesi (consultant). The other authors declare no conflicts of interest.

## Supporting information


**Table S1:** Comparison of characteristics between patients who underwent LT for cirrhosis and HCC.
**Table S2:** Pre‐LT risk associated to overall death after LT in patients transplanted for cirrhosis.
**Table S3:** Pre‐LT risk associated to overall death after LT in patients transplanted for HCC.

## Data Availability

The data that support the findings of this study are available from the corresponding author upon reasonable request.
