# Supplementary material for: Liver Transplantation for HBV‐Related Disease in France: NUC Type Before LT Is Associated With Patient Survival
Source: Liver Int. 2026 Jun 11;46(7):e70737. doi: 10.1111/liv.70737 (PMC13255745; doi:10.1111/liv.70737)
Supplement: Supplementary file 1 — Table S1: Comparison of characteristics between patients who underwent LT for cirrhosis and HCC. Table S2: Pre‐LT risk associated to overall death after LT in patients transplanted for cirrhosis. Table S3: Pre‐LT risk associated to overall death after LT in patients transplanted for HCC. [file LIV-46-0-s001.docx]

**Supplementary Table 1:** Comparison of characteristics between patients who underwent LT for cirrhosis and HCC

|  |  | | Univariate analysis (p) |
| --- | --- | --- | --- |
|  | Cirrhosis (n=435) | HCC (n=526) |  |
| LT period (4 categories) (patients, %) | 105 (24.1) /116 (26.7) /128 (29.4) /86 (19.8) | 135 (25.7) /130 (24.7) /144 (27.4) /117 (22.2) | 0.207 |
| Liver Transplant Center (4 categories) (patients, %) | 27 (6.2) /70 (16.1) /121 (27.8) /217 (49.9) | 23 (4.4) /97 (18.4) /126 (24.0) /280 (53.2) | 0.899 |
| Gender (Male/female) (patients, %) | 352 (80.9) / 83 (19.1) | 432 (82.1) / 94 (17.9) | 0.345 |
| Age (median) | 47 [39-56] | 57 [51-64] | **0.001** |
| MELD score (median) | 20 [14.7-27.1] | 8 [5-14] | **0.0001** |
| CHILD score (median) | C11 [B9-C12] | A6 [A5-B8] | **0.0001** |
| Serum creatinine (median) | 77 [61.5-103.5] | 81 [68-97] | 0.166 |
| BMI at LT listing (median) | 25 [21-27] | 25 [23-28] | 0.205 |
| HBV DNA at LT listing (undetectable/detectable) (patients, %) | 57 (13.1) / 378 (86.9) | 32 (6.1) / 494 (93.9) | 0.435 |
| HDV coinfection (Yes/No) (patients, %) | 52 (12.0) / 383 (88.0) | 69 (13.1) / 457 (86.9) | 0.329 |
| NUC type pre-LT (tenofovir/entecavir/others/none) (patients, %) | 179 (46.0) / 134 (34.4) / 49 (12.6) / 27 (6.9) | 198 (41.1) / 181 (37.6) / 85 (17.6) / 18 (3.7 | 0.251 |

**Supplementary Table 2:** Pre-LT risk associated to overall death after LT in patients transplanted for cirrhosis.

|  | Univariate analysis (p value) | Multivariate analysis | |
| --- | --- | --- | --- |
|  |  | HR (95% CI) | p value |
| LT period (4 categories) | 0.219 |  |  |
| Liver Transplant Center (4 categories) | 0.316 |  |  |
| Gender | 0.349 |  |  |
| Alcohol consumption before LT | 0.482 |  |  |
| Age at LT time | **0.001** | **1.031 (1.002-1.062)** | **0.037** |
| MELD score at LT time | 0.908 |  |  |
| CHILD-PUGH score at LT time | 0.317 |  |  |
| Serum creatinine at LT time | **0.035** | **1.004 (1.001-1.007)** | **0.014** |
| Albumin at LT time | 0.456 |  |  |
| Total bilirubin at LT time | 0.791 |  |  |
| INR at LT time | 0.237 |  |  |
| Natremia at LT time | 0.521 |  |  |
| Overweight | 0.698 |  |  |
| BMI | 0.317 |  |  |
| HBV DNA at LT time (undetectable/detectable) | 0.158 |  |  |
| HBV DNA at LT time (quantifiable versus non-quantifiable) | 0.642 |  |  |
| HBeAg at LT time (pos/neg) | 0.522 |  |  |
| HDV coinfection (Yes/No) | **0.022** | 1.002 (0.542-1.852) | 0.995 |
| Pre-LT NUC type (tenofovir versus entecavir) | **0.040** | 1.270 (0.651-2.476) | 0.483 |

Abbreviations: BMI: Body-Mass Index; DNA: Deoxyribonucleic Acid; HBV: Hepatitis B; HDV: Hepatitis D; INR: International Normalized Ratio; LT: Liver Transplantation; NUC: nucleos(t)ide analogues; MELD: Model for End-Stage Liver Disease;

**Supplementary Table 3:** Pre-LT risk associated to overall death after LT in patients transplanted for HCC.

|  | Univariate analysis (p value) | Multivariate analysis | |
| --- | --- | --- | --- |
|  |  | HR (95% CI) | p value |
| LT period (4 categories) | 0.547 |  |  |
| Liver Transplant Center (4 categories) | 0.279 |  |  |
| Gender | 0.248 |  |  |
| Alcohol consumption before LT | 0.449 |  |  |
| Age at LT time | **0.002** | **1.030 (1.006-1.054)** | **0.013** |
| MELD score at LT time | 0.363 |  |  |
| CHILD-PUGH score at LT time* | **0.0001*** | 0.933 (0.846-1.029) | 0.168 |
| Serum creatinine at LT time* | **0.035*** | 0.999 (0.996-1.003) | 0.748 |
| Albumin at LT time* | **0.041*** |  |  |
| Total bilirubin at LT time* | **0.226*** |  |  |
| INR at LT time* | **0.032*** |  |  |
| Natremia at LT time | 0.191 |  |  |
| AFP score (<2 vs ≥2) | 0.592 |  |  |
| Overweight | 0.930 |  |  |
| BMI | 0.180 |  |  |
| HBV DNA at LT time (undetectable/detectable) | 0.831 |  |  |
| HBV DNA at LT time (quantifiable versus non-quantifiable) | 0.979 |  |  |
| HBeAg at LT time (pos/neg) | 0.143 |  |  |
| HDV coinfection (Yes/No) | 0.414 |  |  |
| Pre-LT NUC type (Tenofovir versus entecavir) | **0.060** | **1.699 (1.120-2.578)** | **0.013** |

Abbreviations: ALF: Acute Liver Failure; BMI: Body-Mass Index; DNA: Deoxyribonucleic Acid; HBV: Hepatitis B; HDV: Hepatitis D; HCC: Hepatocellular Carcinoma; INR: International Normalized Ratio; LT: Liver Transplantation; NUC: nucleos(t)ide analogues; MELD: Model for End-Stage Liver Disease;

*Due to multicollinearity, the multivariate analysis model only took into account uncorrelated variables: Several variables were collinear with the LT indication: MELD score, CHILD score, biological parameters taken into account by these scores. We chose the LT indication for the multivariate model.
